# Hydrophobia

**Published:** 1869-11

**Authors:** 


### HYDROPHOBIA.

The Philadelphia *North American* says, "A little girl, twelve years of age, living in the Nineteenth Ward, was nipped in the finger by a dog on the afternoon of July 12th. Her name was Georgiana McCready. Three days ago she was seized by the premonition of the hydrophobia. The most distinguished of the profession of medicine were called to her rescue. By their sanction, with that of friends, relatives, and all to whom the life of the child was dear, her sufferings were stayed by the merciful interposition of poison."

We transfer the foregoing painful paragraph to the columns of the JOURNAL, for the opportunity of saying that in the town of Norfolk, Litchfield County, Connecticut, a family of physicians, the Doctors Welch, are said to have treated many cases of hydrophobia with remarkable success; having lost, as has been represented, but one patient in many years—and that one dying because the strength had been exhausted from the agonizing spasms; but even in this case the benefits of the treatment were most convincing.

The Doctors Welch cheerfully impart their information to educated physicians without charge, and it may answer a good purpose to make this announcement, for the reason that there is evidence to believe that hydrophobic symptoms may arise from nervousness or apprehension, and, by consulting persons in whom confidence is reposed, the mind may be relieved and restored to its healthful condition. The practice pursued is understood to have once been published by legislative uthority, the -knowledge of it having been purchased by the State of New York. If this is the case, some one would be doing a public good by searching the public records at Albany, and republishing the documents.

